# Case report of a cervical myelomalacia caused by a thoracolumbar intradural disc herniation leading to intracranial hypotension

**DOI:** 10.1007/s00415-020-10247-1

**Published:** 2020-10-03

**Authors:** M. Ueberschaer, M. Patzig, K. Mueller, J. Schwarting, R. Trabold, J.-C. Tonn

**Affiliations:** 1 Department of Neurosurgery, University Hospital, LMU Munich, Marchioninistr. 15, 81377 Munich, Germany; 2 Institute of Neuroradiology, University Hospital, LMU Munich, Munich, Germany; 3 Department of Neurology, University Hospital, LMU Munich, Munich, Germany

**Keywords:** Case report, Intradural disc herniation, Intracranial hypotension, Myelomalacia, CSF leak

## Abstract

A 50-year-old patient was admitted with symptoms of intracranial hypotension. MRI revealed a cervical myelomalacia caused by engorged epidural veins leading to a stenosis of the spinal canal. This condition is rarely described in patients with hydrocephalus and ventricular shunts suffering from chronic overdrainage. However, the reason in this patient was a CSF leak caused by an intradural disc herniation at T12/L1. After surgery, symptoms resolved and the cervical myelomalacia and the swollen epidural veins disappeared on postoperative MRI. In patients with engorged cervical epidural veins without a ventricular shunt, a CSF leak has to be considered.

## Introduction

Intradural disc herniation (IDH) is a rare entity accounting for 0.26–0.3% of all disc herniations [1, 4, 7]. They typically occur at the lower lumbar spine (92%) and are usually associated with neurological deteriorations and/or local pain [3, 6, 7, 8, 12]. Here, we present the case of a 50-year-old patient who was admitted merely with orthostatic headache due to a CSF leak caused by an IDH at the level of T12/L1 with radiological signs of a cervical myelomalacia caused by an engorged epidural venous plexus.

## Results

### Patient Information and clinical findings

A 50-year-old female patient was admitted with a 7-week history of postural headache deteriorating in upright position accompanied by nausea and vomiting. At neurological examination, she presented without any pathological findings, especially no symptoms of lumbar nerve root compression. The medical history of the athletic patient revealed no relevant previous disease or trauma.

### Diagnostic assessment

**Fig. 1:**
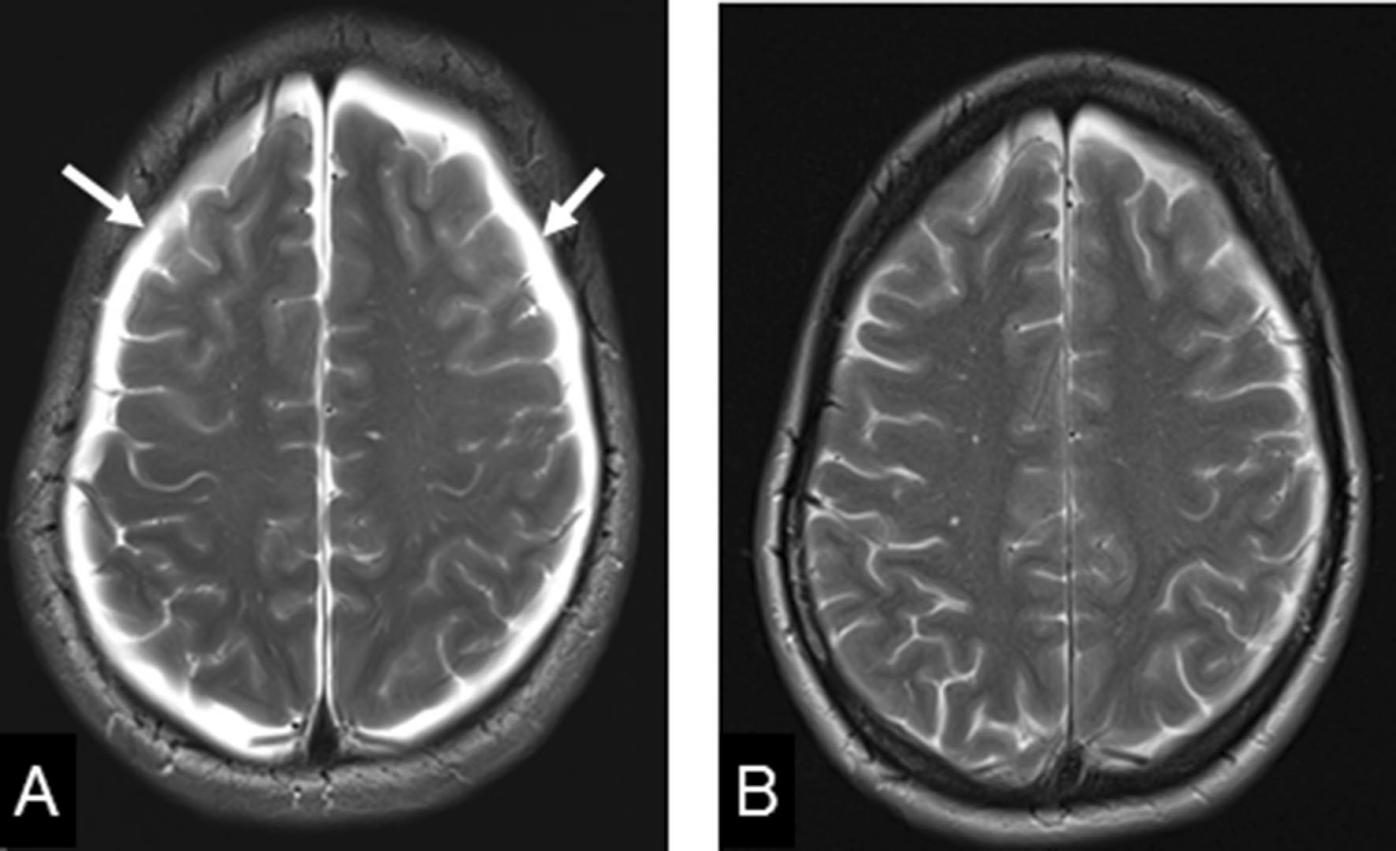
**a** Bilateral subdural hygromas in T2 weighted MRI (arrow); **b** significantly improved hygromas according to postoperative MRI

**Fig. 2:**
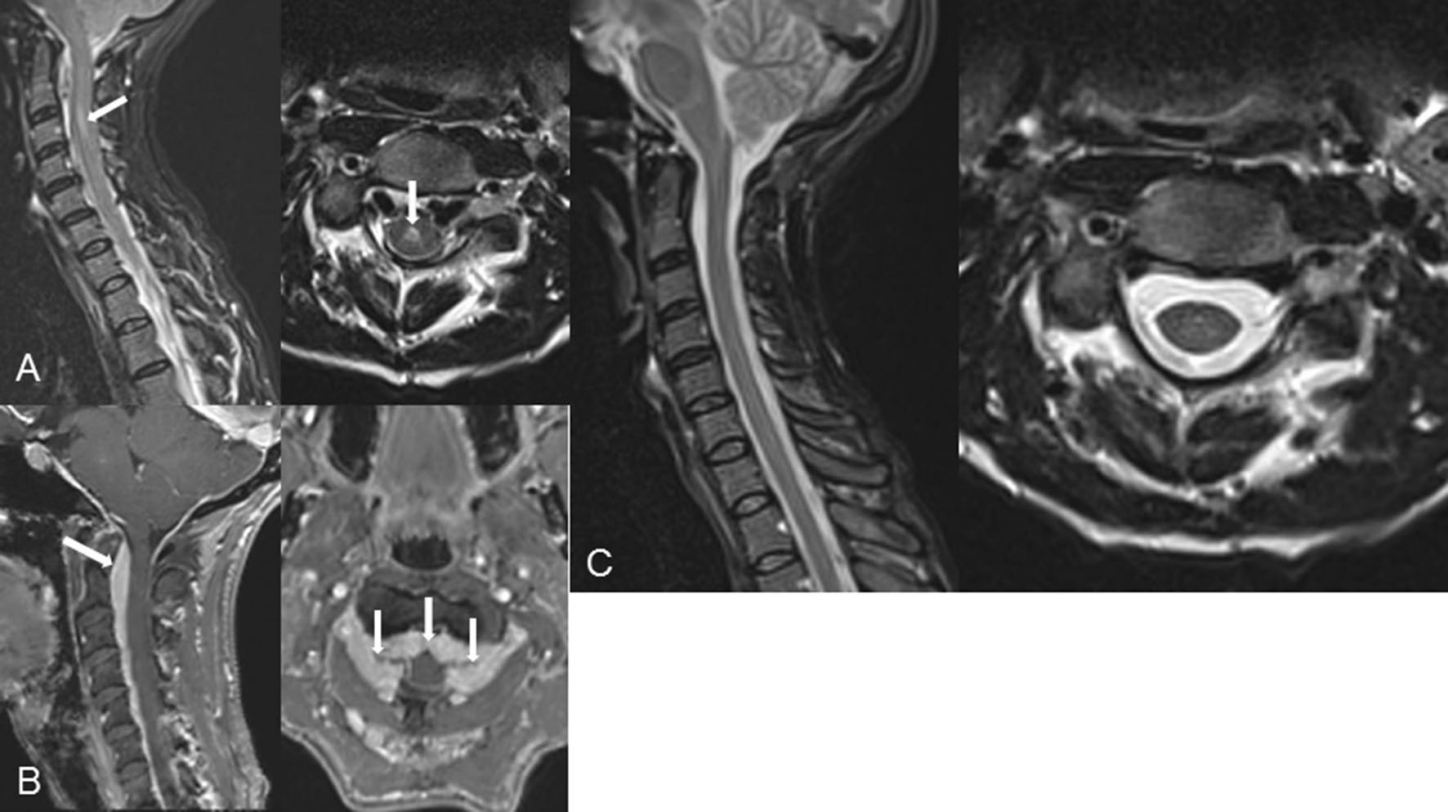
**a** Preoperative myelomalacia (arrows) at C3 in sagittal STIR-sequences and axial T2 weighted MRI; **b** Preoperative CE-MRI showing the prominent epidural veins leading to a spinal cord compression (arrows); **c** Normal epidural space without evidence of persisting myelomalacia at C3 according to the sagittal postoperative STIR- and axial T2 weighted MRI

**Fig. 3:**
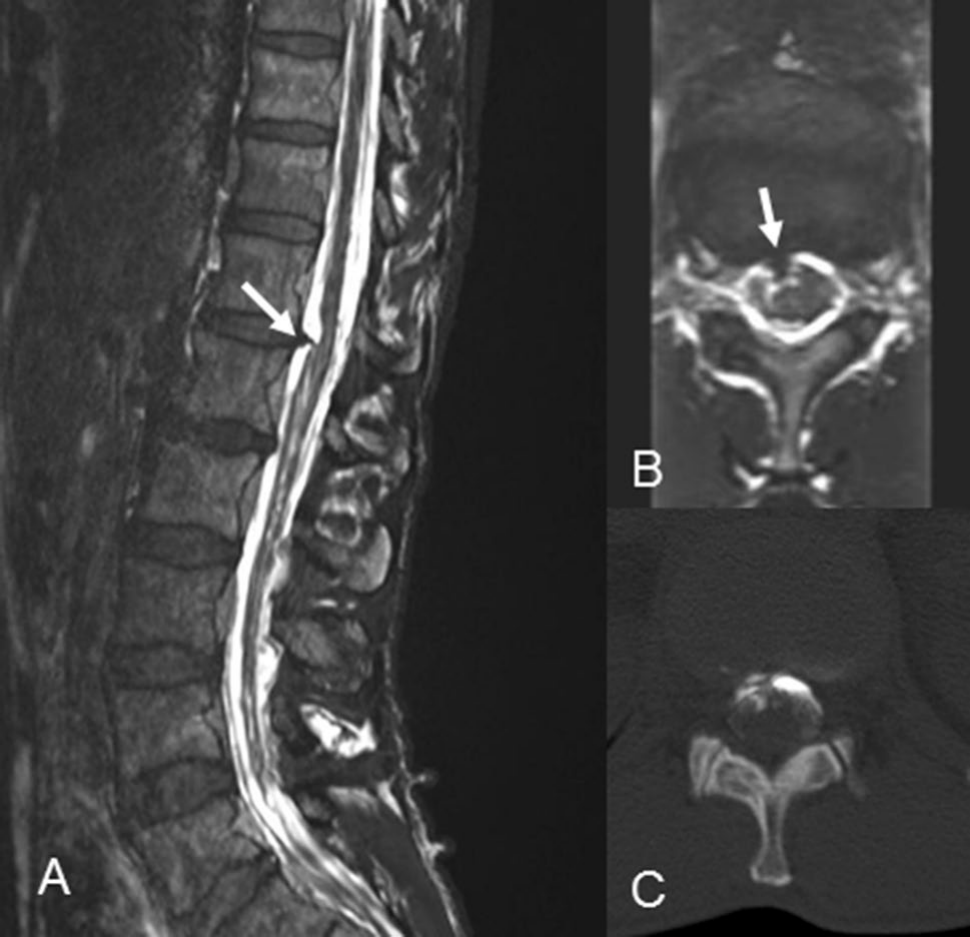
**a, b** Sagittal and axial CISS MRI shows the discogenic spur at T12/L1(arrow); **c** CT-myelography with epidural contrast-pooling and right-sided ventromedial contrast agent extravasation

Cranial magnetic resonance imaging (MRI) disclosed bilateral subdural hygromas typical for intracranial hypotension (Fig. 1). MRI of the cervical spine showed massively engorged epidural veins leading to a severe stenosis of the spinal canal with concurrent signs of myelomalacia at C3 (Fig. 2a, b). MRI of the lumbar spine presented a prominent disc spur at the level of T12/L1. Additional CT-myelography revealed a CSF leakage at the right ventral circumference of the dural sac correlating with the herniated disc (Fig. 3).

### Clinical course

Initial treatment with caffeine did not lead to an improvement of the symptoms. A lumbar puncture with injection of a blood patch was not performed due to the bilateral hygromas. Therefore, surgery was indicated.

**Fig. 4:**
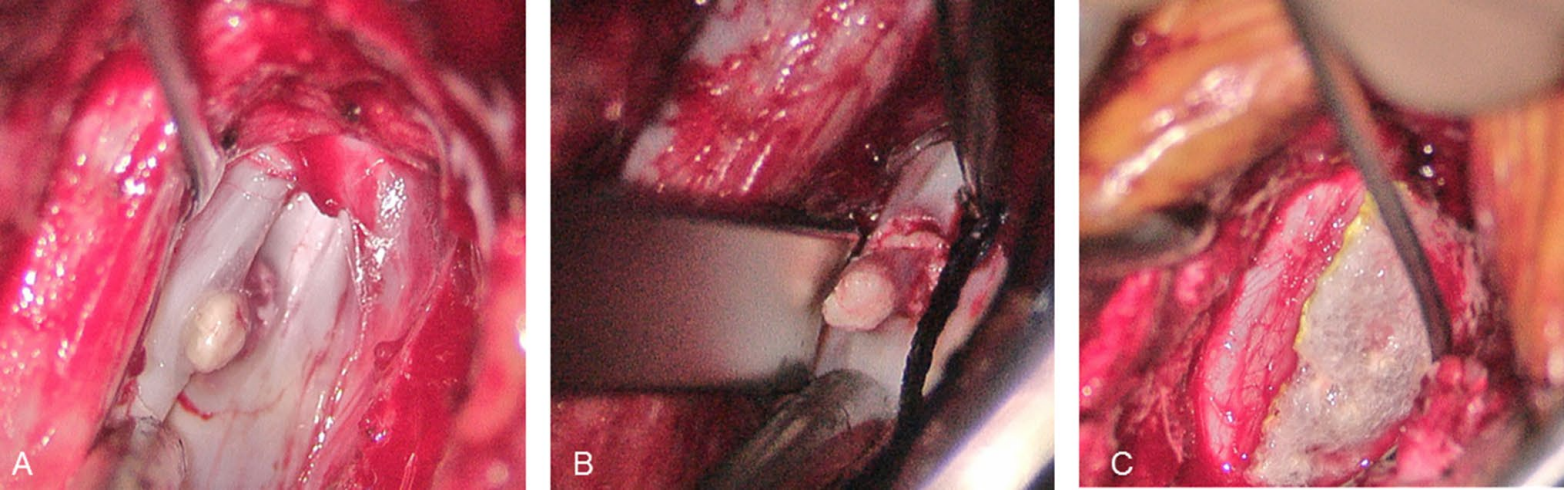
**a** Discogenic spur perforating the dural sac; **b** the spur after seperation from the dura; **c** final result after dural suture and application of a fibrin sponge (Tachosil)

After microsurgical right-sided fenestration at the level T12/L1, the herniated disc spur was identified perforating the posterior longitudinal ligament and the dura with pronounced CSF leakage. The spur was carefully detached from the dural sack under the microscope. After removal, the dural defect was sutured and secured with a fibrin sponge (Tachosil^®^) (Fig. 4). After bed rest for 72 h, the headache resolved completely.

MRI 3 days after surgery showed resolution of both the hygroma and the myelomalacia at C3. The width of the cervical epidural space returned to normal due to the regression of the engorged peridural veins (Fig. 2c).

Histopathological examination confirmed a partially calcified disc herniation.

The patient was discharged at day 5 after the surgery without postural headaches or any neurological deficits. At the 3-month follow-up, the patient still had no orthostatic headache and the cranial MRI as well as the MRI of the cervical spine showed no pathological findings.

## Discussion

Regarding the surgical procedure, resection of the herniated disc and suture of the dural leakage were feasible via an interlaminary fenestration and a more invasive transdural approach could be avoided. The intraoperative finding matched with the report of Beck et al. who identified discogenic microspurs as origin for a majority of CSF leaks in their case series [2].

In addition to the scant literature describing intracranial hypotension caused by an IDH [5], our case additionally presented a cervical myelomalacia at the level of C3 caused by an engorged epidural venous plexus. This rare phenomenon has been observed in intracranial hypotension caused by chronic overdrainage in patients with hydrocephalus and ventricular shunts [9, 10, 13–15]. In these cases, a mechanism is assumed which leads to an imbalance of intracranial fluids [14]. In accordance to the Monro–Kellie hypothesis, the total volume of intracranial blood, CSF and brain tissue remains constant. Any reduction of one of these components leads to an excess of one or more of the other components [11]. In our patient, the reduction of CSF volume and pressure caused an increase of the intravasal blood volume (the brain tissue volume remaining constant). The increased intracranial blood volume in this instance mainly affects the venous system with subsequent rise of the venous pressure leading also to an engorgement of the blood volume in the epidural venous plexus of the cervical spine. The dilation of the veins not only compensates the lack of CSF volume, but also leads to a compression of the spinal cord. The dilation of the veins is, thus, at the expense of the volume of the spinal cord explaining the spinal cord edema. Such a condition, previously reported in the context of intracranial hypotension, has never been described in a patient with CSF leak caused by a herniated disc. The fact that both headache and radiological signs of myelomalacia with engorged epidural veins resolved immediately and concomitantly after closure of the CSF leak underlines this concept of pathophysiology.

Although patients usually have orthostatic headaches that primarily lead to diagnosis, CSF leakage must be considered in patients with swollen epidural veins of the cervical spine without a ventricular shunt. Dilation of the venous plexus appears to cause potential damage due to the continuous compression of the spinal cord according to MRI. The absence of pain and neurological deficits does not rule out a disc herniation as the underlying cause.
